# Organ Preservation in Esophageal Cancer: Current Strategies, Challenges, and Future Directions

**DOI:** 10.3390/cancers17213559

**Published:** 2025-11-03

**Authors:** Wenyi Liu, Baihua Zhang, Chunguang Wang, Xin Yu, Longde Du, Zhentao Yu, Mingqiang Kang

**Affiliations:** 1 Department of Thoracic Surgery, Fujian Medical University Union Hospital, Fuzhou 350001, China; wyliu1@126.com; 2 Department of Thoracic Surgery, National Cancer Center/National Clinical Research Center for Cancer/Cancer Hospital & Shenzhen Hospital, Chinese Academy of Medical Sciences and Peking Union Medical College, Shenzhen 518116, China; baihuaz126@126.com (B.Z.); wangchunguang@chcamssz.ac.cn (C.W.); yuxin@chcamssz.ac.cn (X.Y.); dulongde@chcamssz.ac.cn (L.D.); 3 Department of Thoracic Surgery, Hunan Cancer Hospital and the Affiliated Cancer Hospital of Xiangya School of Medicine, Central South University, No. 283 Tongzipo Road, Changsha 410013, China

**Keywords:** esophageal cancer, esophageal squamous cell carcinoma, esophageal adenocarcinoma, organ preservation, chemoradiotherapy, active surveillance, immunotherapy, clinical trials, biomarkers, proton therapy

## Abstract

**Simple Summary:**

Esophageal cancer is a serious disease affecting over 600,000 people worldwide each year. Traditionally, surgery to remove the esophagus has been the main treatment, but it can cause major side effects like trouble swallowing or eating. This review looks at newer ways to treat the cancer without surgery, called “organ preservation.” These include chemotherapy combined with radiation, and sometimes immunotherapy drugs. Studies show these methods can work as well as surgery for some patients, helping them keep their esophagus and improve quality of life. However, challenges like cancer coming back or side effects remain. Future advances, like better tests for early detection and personalized treatments, could make these options even better. This review may help doctors choose the right approach for each patient.

**Abstract:**

Esophageal cancer (EC) continues to pose a major global health burden, ranking as the ninth most common malignancy and sixth leading cause of cancer mortality, with over 600,000 new cases and 500,000 deaths annually as of 2025. While esophagectomy has long been the standard for curative intent in resectable disease, organ preservation strategies have advanced significantly, offering viable alternatives for patients with locally advanced esophageal squamous cell carcinoma (ESCC) or those unsuitable for surgery due to comorbidities. These approaches encompass definitive chemoradiotherapy (dCRT), neoadjuvant chemoradiotherapy (nCRT) followed by active surveillance (“watch-and-wait”), and innovative integrations of immunotherapy and targeted therapies. This narrative review synthesizes evidence from recent clinical trials, systematic reviews, and international guidelines up to 2025, demonstrating that organ-sparing protocols can achieve comparable overall survival (OS) rates—often exceeding 50% at 5 years in selected cohorts-while substantially enhancing quality of life (QoL) by preserving esophageal function. For instance, the SANO trial (2025) confirmed non-inferiority of active surveillance post-nCRT, with 2-year OS of 74% versus 71% for standard surgery. Key challenges include imprecise response assessment, locoregional recurrences (20–30%), and treatment-related toxicities such as esophageal strictures. Emerging trials like ESOSTRATE and PALACE3 are evaluating immunotherapy-enhanced regimens, potentially expanding organ preservation to esophageal adenocarcinoma (EAC). With genomic biomarkers and novel modalities like proton therapy, personalized organ preservation promises to broaden applicability, reduce morbidity, and improve outcomes across histological subtypes. Additionally, recent studies emphasize the role of liquid biopsies, such as circulating tumor DNA (ctDNA), in monitoring treatment response and guiding surveillance, potentially reducing the need for invasive procedures and improving detection of minimal residual disease. The aim of this review is not only to summarize recent trials but to synthesize them into an operational framework that clinicians and researchers can apply: a decision algorithm for selecting organ preservation candidates. This is the novel element that distinguishes this work from prior narrative reviews.

## 1. Introduction

Esophageal cancer (EC) remains a formidable challenge in oncology, with an estimated incidence of over 600,000 cases worldwide in 2025, reflecting a slight increase driven by rising obesity and gastroesophageal reflux disease (GERD) in Western countries [1]. The disease encompasses two main histological subtypes: esophageal squamous cell carcinoma (ESCC), predominant in Asia and linked to tobacco and alcohol use, and esophageal adenocarcinoma (EAC), increasingly common in the West due to Barrett’s esophagus [2]. Despite multimodal advancements, 5-year survival rates hover around 20–25% globally, underscoring the need for innovative treatments that balance efficacy with patient-centered outcomes.

The natural history of EC typically begins asymptomatically in its early stages, often detected incidentally or through screening in high-risk populations. For ESCC, the disease frequently originates from squamous dysplasia in the mid-to-upper esophagus and progresses rapidly to invasive carcinoma, leading to symptoms such as dysphagia, odynophagia, weight loss, and hoarseness as the tumor obstructs or invades adjacent structures. In contrast, EAC often develops from Barrett’s esophagus, a metaplastic change due to chronic GERD, progressing through low- and high-grade dysplasia to adenocarcinoma in the distal esophagus or gastroesophageal junction. This progression can take years, but once invasive, it metastasizes early to lymph nodes and distant sites, contributing to poor prognosis if not detected early [3].

Historically, esophagectomy was the curative mainstay, but its associated perioperative mortality and long-term complications-such as dysphagia, reflux, and nutritional deficits-have prompted a shift toward organ preservation. This approach aims to eradicate disease while maintaining esophageal integrity, inspired by successes in laryngeal and rectal cancers [4]. Organ preservation is defined as non-surgical strategies that prioritize functional esophageal retention without compromising survival. Treatment choices for EC are stage-dependent and multidisciplinary. For early-stage (T1N0) disease, endoscopic resection or ablation, including photodynamic therapy, is preferred for superficial lesions. In locally advanced disease (T2–4 or N+), options include neoadjuvant chemoradiotherapy (nCRT) followed by surgery (trimodality therapy) as per the CROSS protocol, or definitive chemoradiotherapy (dCRT) for non-surgical candidates. Advanced or metastatic disease relies on systemic therapies like chemotherapy combined with immunotherapy (e.g., PD-1 inhibitors). Organ preservation strategies, such as dCRT or nCRT with active surveillance, are increasingly selected for patients with good performance status and favorable response predictors, aiming to avoid surgery’s morbidity while achieving comparable outcomes. This review narrates the evolution of organ preservation in EC, drawing from literature through mid-2025, including pivotal trials and emerging therapies, to guide clinical practice and highlight research gaps. Furthermore, advancements in molecular profiling and imaging techniques are enabling more precise patient selection, with ongoing research focusing on integrating artificial intelligence (AI) for response prediction and risk stratification.

## 2. Methods

This narrative review was conducted by searching databases including PubMed, Embase, Cochrane Library, and ClinicalTrials.gov for articles published from 2015 to September 2025. Keywords included “esophageal cancer,” “organ preservation,” “chemoradiotherapy,” “active surveillance,” and “immunotherapy.” We prioritized randomized controlled trials (RCTs), prospective cohorts, and systematic reviews, while excluding case reports, non-English literature, and studies with sample sizes under 20 or lacking peer review. Inclusion criteria focused on studies demonstrating clinical outcomes (e.g., OS, DFS), with emphasis on human trials; exclusion criteria included animal studies. We selected over 100 sources for synthesis, ensuring comprehensive coverage. No formal meta-analysis was performed, focusing instead on qualitative integration of evidence to provide a comprehensive overview. To ensure comprehensiveness, additional searches were performed for terms such as “proton therapy,” “biomarkers,” “immunotherapy combinations,” and “future directions in esophageal cancer,” incorporating recent 2025 publications and trial updates from sources like ASCO and ESMO conferences. The search was systematic, with duplicate removal using EndNote software (X9.3.3), and two authors independently reviewed abstracts for relevance.

## 3. Key Content and Findings

### 3.1. Historical Perspective and Rationale for Organ Preservation

The pursuit of organ preservation in EC mirrors paradigms in other sites, where non-surgical modalities preserve function without survival compromise. Early 20th-century efforts relied on radiotherapy alone, yielding dismal 5-year OS (10–20%) due to poor local control. The integration of chemotherapy in the 1980s marked a turning point; the RTOG 85-01 trial (1999) demonstrated concurrent CRT superiority over radiation monotherapy, with 26% 5-year OS in locally advanced disease [5].

The landmark CROSS trial (2012, long-term follow-up 2021) established nCRT (carboplatin/paclitaxel + 41.4 Gy) followed by surgery as standard, improving median OS from 24 to 81 months in survivors [6]. Retrospective data suggested dCRT could match trimodality outcomes in ESCC, prompting selective surgery approaches [7]. However, the CROSS trial’s evidence is limited by its relatively small sample size (*n* = 366) and heterogeneous population, with conflicting results in subgroup analyses for EAC vs. ESCC, highlighting the need for histology-specific evaluations.

A retrospective study from 2015 to 2020 in two tertiary centers [8] included 41 patients with early esophageal cancer who had histologically complete resection but with poor differentiation, lymphovascular invasion, or deep submucosal invasion. After endoscopic resection, 13 patients (32%) were closely monitored, and 28 (68%) were treated with chemoradiotherapy or radiotherapy alone. At the end of the follow-up, the disease-free survival (DFS), overall survival (OS), and cancer-specific survival (CSS) in the close-follow-up group were 92%, 92%, and 100%, respectively, compared to 75%, 79%, and 96% in the chemoradiotherapy group. Serious adverse events related to chemoradiotherapy occurred in 10% of the patients, and there were no treatment-related deaths. This study indicates that close follow-up may be a viable alternative to systematic esophagectomy in such cases.

In the context of rectal cancer, which shares some similarities in organ preservation concepts, total neoadjuvant therapy (TNT) has shown improved oncological outcomes and organ preservation rates [9]. A historical cohort study compared the quality of life (QoL) of post-TNT watch-and-wait (W&W) patients to a historical cohort of patients who underwent standard care. Questionnaires were completed by 29 of 41 patients in the pTNT W&W group and 33 of 63 patients in the STD group. The pTNT W&W group had significantly lower rates of lower anterior resection syndrome (LARS) (55.6% vs. 87.5%, *p* = 0.012) and fewer cases of major LARS (29.6% vs. 58.3%, *p* = 0.039). They also had significantly improved QoL scores across several parameters of EORTC-QLQ-CR29. These findings suggest that organ-preserving approaches can not only preserve the organ but also improve the patient’s QoL, which has gradually influenced the treatment paradigm in esophageal cancer as well. Nonetheless, these rectal cancer analogies have limitations due to differing tumor biology, with inconsistent evidence when applied to EC.

By 2025, guidelines from NCCN, ESMO, and ASCO endorse dCRT for cervical ESCC and as an alternative for operable patients preferring preservation [10,11], reflecting a paradigm shift toward QoL optimization. Building on this, the ESOPEC trial (2025 updates) has explored perioperative chemotherapy regimens like FLOT versus CROSS, showing potential benefits in EAC, while highlighting the need for tailored approaches based on histology [12]. The ESOPEC trial, however, faces criticism for potential selection bias in its multicenter design.

### 3.2. Current Organ Preservation Strategies

To clearly delineate these strategies, we categorize them into definitive, neoadjuvant, endoscopic therapies and integrated approaches.

#### 3.2.1. Definitive Chemoradiotherapy (dCRT)

dCRT remains foundational for unresectable locally advanced EC, combining platinum-based agents with 50–66 Gy radiation, achieving 5-year OS of 30–45% in ESCC [13]. Common regimens include cisplatin plus 5-fluorouracil (5-FU) or carboplatin/paclitaxel, delivered concurrently with external beam radiation. Patient selection typically favors those with good performance status (ECOG 0–2), no distant metastases, and tumors amenable to radiation fields, such as mid-esophageal lesions. Treatment-related toxicities are significant, including acute esophagitis, pneumonitis, and late complications like strictures or fistulas, which can impact QoL [14]. Modern techniques like intensity-modulated radiotherapy (IMRT) and proton beam therapy (PBT) enhance locoregional control (>70%) while minimizing cardiac and pulmonary toxicity. For early-stage (T1–2N0) disease, endoscopic resection (ER) plus adjuvant CRT offers 80–90% DFS with low morbidity, as evidenced in 2020 cohorts [8]. Data from the NRG-GI006 trial compare proton beam therapy (PBT) versus intensity-modulated radiation therapy (IMRT), suggesting that for locally advanced esophageal cancer, PBT reduced the risk and severity of AEs compared with IMRT while maintaining similar PFS [15]. Meta-analyses did not demonstrate a clear survival advantage for surgery-based trimodality therapy over dCRT, these results support dCRT’s efficacy [16]. Non-surgical treatment for esophageal cancer may become part of future personalized and tailored therapeutic approaches. However, to date, there remains a lack of conclusive evidence proving its non-inferiority compared to surgical treatment. Heterogeneity in radiation doses and chemotherapy regimens across studies limits direct comparisons, and long-term follow-up data indicate higher locoregional recurrence rates with dCRT alone.

#### 3.2.2. Neoadjuvant Therapy Followed by Active Surveillance

Active surveillance post-nCRT involves multimodal monitoring (endoscopy, biopsies, PET-CT) to detect residual disease, reserving esophagectomy for non-responders [17]. The preSANO trial (2018) validated clinical complete response (cCR) assessment with 80% accuracy [18]. The CROC trial (2024) confirmed dCRT viability for downstaged remarkable responders, with 83.7% 3-year OS [19]. The SANO trial’s full 2025 publication show that active surveillance allowed 91 of the 198 participants achieving clinical complete response to avoid an unnecessary oesophagectomy [20]. Limitations include the SANO trial’s focus on ESCC-dominant cohorts, with potential under-detection of residual disease in 10–20% of cases due to imaging inaccuracies.

#### 3.2.3. Endoscopic Therapies for Early-Stage Disease

For superficial or early-stage EC (T1a or high-grade dysplasia in Barrett’s esophagus), endoscopic therapies represent a cornerstone of organ preservation, minimizing the need for more invasive interventions. Photodynamic therapy (PDT) is a notable example, involving the administration of a photosensitizing agent (e.g., porfimer sodium) followed by endoscopic light activation to induce tumor necrosis through reactive oxygen species. PDT is particularly effective for Barrett’s esophagus with high-grade dysplasia or early EAC [21]. It is also used in early ESCC, often in combination with endoscopic mucosal resection (EMR) for deeper lesions. Advantages include low morbidity, outpatient feasibility, and preservation of esophageal function, with common side effects limited to transient photosensitivity and esophageal strictures. However, challenges include variable penetration depth, potential for incomplete response in submucosal invasions, and recurrence, necessitating surveillance. Critical evaluation notes small cohort sizes in early studies, but meta-analyses confirm its role in high-risk populations, broadening organ preservation options beyond CRT [22].

#### 3.2.4. Integration of Immunotherapy and Targeted Therapies

Immunotherapy has revolutionized EC management, with PD-1/PD-L1 inhibitors like pembrolizumab and nivolumab approved for advanced disease [23]. In the phase III CheckMate 577 trial (2021), patients with resected stage II/III esophageal or gastroesophageal junction cancer who had residual disease after trimodality therapy (neoadjuvant chemoradiotherapy followed by surgery) experienced a near doubling of median disease-free survival with adjuvant nivolumab compared with placebo (22.4 vs. 11.0 months; HR = 0.69; *p* < 0.001). Importantly, by delaying or preventing recurrence, this strategy supports the broader paradigm of organ preservation, as effective systemic control may reduce the need for further mutilating interventions and help maintain postoperative quality of life. Early-phase studies such as PALACE-1 have reported pathologic complete response (pCR) rates up to 55.6% with neoadjuvant CRT plus pembrolizumab in ESCC. While PALACE-2 has been proposed to further evaluate this approach [24,25]. A 2024 ASCO GI abstract (TPS431) reported that the FLOT-AIO (PRESTO) phase II trial is actively investigating a durvalumab-based organ preservation strategy in early-stage, resectable EAC (cT1–T2N0), combining durvalumab with FLOT-based chemotherapy and chemoradiation, aiming to avoid radical surgery by achieving high clinical/pathological complete response rates (targeting ≥ 75%) [26]. According to its ClinicalTrials.gov entry (NCT05713838), the design includes initial induction with durvalumab + FLOT, followed by durvalumab + mFOLFOX with concurrent radiation (50 Gy), with cCR/pCR assessed at 8 weeks to determine whether surgery can be safely omitted in responders [27]. However, these early-phase trials suffer from small samples (e.g., PALACE-1 *n* = 18), limiting robust conclusions.

Targeted therapies, such as the ToGA trial (Trastuzumab for Gastric Cancer), was a pivotal phase III study that established the efficacy of adding trastuzumab to chemotherapy in patients with HER2-positive advanced gastric or gastroesophageal junction (GEJ) adenocarcinoma. The trial demonstrated a significant improvement in overall survival (OS) and progression-free survival (PFS) with the addition of trastuzumab to standard chemotherapy [28]. A phase II study in locally advanced gastric and gastroesophageal adenocarcinoma combined FLOT with trastuzumab reported pCR rate of 21.4%, with an additional 25% achieving near-complete responses [29]. In October 2024, the U.S. Food and Drug Administration (FDA) approved zolbetuximab (Vyloy) in combination with fluoropyrimidine- and platinum-based chemotherapy as a first-line treatment for patients with locally advanced unresectable or metastatic HER2-negative, claudin 18.2-positive gastric or gastroesophageal junction (GEJ) adenocarcinoma. This approval was based on results from the SPOTLIGHT and GLOW phase III clinical trials [30,31], which demonstrated that zolbetuximab plus chemotherapy significantly improved overall survival and progression-free survival compared to chemotherapy alone. These findings suggest that zolbetuximab may also benefit gastric-type EAC patients, potentially enabling more individuals to opt for surveillance over surgery. However, further clinical trials and studies are needed to confirm these benefits in EAC specifically. Critical evaluation notes the ToGA trial’s focus on GEJ, with limited extrapolation to pure EC due to biological differences.

### 3.3. Key Clinical Trials and Evidence

The following table summarizes pivotal trials, updated with 2025 data: Table 1.

These trials underscore the non-inferiority of surveillance in responders, particularly ESCC, with SANO providing level 1 evidence for broader adoption. The addition of ESOPEC and NRG-GI006 highlights histology-specific strategies and radiation advancements, respectively. In addition to CROSS, the JCOG1109 trial evaluated preoperative chemotherapy (e.g., docetaxel/cisplatin/5-FU) followed by surgery in ESCC, achieving 3-year OS of 72.1%, emphasizing surgery’s local control benefits but with higher morbidity compared to preservation approaches.

## 4. Discussion

The landscape of EC treatment is rapidly evolving, with a strong emphasis on organ preservation strategies that minimize invasive procedures while maximizing patient outcomes. Organ preservation offers equivalent oncologic outcomes to surgery in select EC patients, with superior QoL metrics such as reduced dysphagia and better nutritional status [14,20]. However, challenges abound: response assessment accuracy is limited (PET-CT/endoscopy miss 10–20% residual disease) [37,38], leading to recurrences in 20–30% of cases, often requiring salvage surgery with heightened risks [39]. Toxicities like strictures (10–15%), pericarditis, and rare complications (e.g., small bowel metastasis) persist, necessitating multidisciplinary management [40]. Histology influences success-ESCC responds better than EAC-while patient factors like performance status are crucial [41]. Compared to historical data, 2025 immunotherapy integrations have boosted cCR rates, but long-term data are needed. Additional challenges include access disparities to advanced therapies like proton therapy, which is limited to specialized centers [42], and the psychological burden of surveillance on patients, with anxiety rates reported up to 40% in cohort studies [43]. Moreover, resistance mechanisms to immunotherapy, such as tumor microenvironment alterations, pose barriers, as evidenced in 2025 biomarker analyses showing PD-L1 expression variability [44]. SEER-based analyses have shown that patients with lower income or socioeconomic status are less likely to receive endoscopic or organ-preserving therapies and experience poorer cancer-specific and overall survival, indicating that despite rising organ preservation rates, disparities in outcomes persist across socioeconomic groups [45,46].

### Clinical Implications

**Figure 1:**
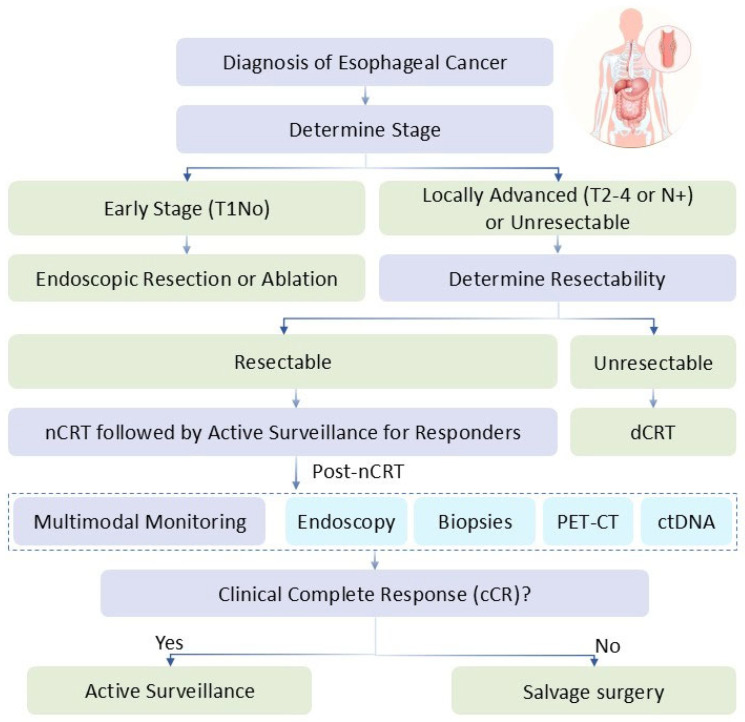
Conceptual Diagram of Treatment Algorithm—A flowchart starting with diagnosis, branching to nCRT/dCRT based on stage, followed by surveillance for cCR or surgery for non-responders.

Organ preservation can be integrated into treatment algorithms via multidisciplinary tumor boards, with patient selection refined by biomarkers (e.g., ctDNA), performance status (ECOG 0–1) and the other screening methods as depicted in the treatment algorithm (Figure 1). Clinicians should anticipate benefits like improved QoL but risks such as 20–30% locoregional recurrences, which reduce OS by 10–20%. Surgery provides superior local control, but preservation avoids morbidity in responders. The JCOG1109 outcomes underscore preoperative chemotherapy’s role, but direct comparisons favor preservation in selected ESCC cases for QoL gains.

## 5. Future Directions

Genomic advancements, such as PD-L1 expression and microsatellite instability (MSI) testing [47], are central to identifying patients suitable for immunotherapy-based preservation. Studies highlight how these biomarkers predict tumor response, with PD-L1 often correlating with better outcomes in immune checkpoint inhibitor therapies. For instance, MSI-high tumors in EC subsets show enhanced responsiveness, though broader validation is needed across diverse populations. Circulating tumor DNA (ctDNA) [48] emerges as a dynamic biomarker, offering real-time monitoring of tumor burden and treatment response. Meta-analyses indicate that ctDNA detection correlates with poorer overall and progression-free survival, positioning it as a tool for personalized selection in preservation protocols. Challenges include assay sensitivity and standardization, but its non-invasive nature broadens applicability. Feasibility is high for ctDNA in urban centers, but limitations like cost and false positives prioritize it for high-risk patients.

Novel treatment modalities are addressing toxicity concerns in EC radiotherapy. Proton therapy demonstrates superior dosimetric advantages, reducing hematologic and cardiopulmonary toxicities compared to photon-based approaches [49]. Clinical studies report lower risks of adverse events, with survival benefits in select cohorts. Hypofractionated regimens, delivering higher doses in fewer sessions, have shown safety and efficacy in palliative and curative settings, with comparable survival to conventional fractionation at reduced costs. Ongoing investigations focus on integrating these with chemotherapy for localized EC, though long-term data on recurrence rates are pending [50]. Current limitations include proton therapy’s limited availability; prioritization should focus on randomized trials for evidence.

Preclinical models like organoids [51] and esophageal organ chips [52] are revolutionizing drug screening and response prediction. Organoids mimic EC heterogeneity, enabling high-throughput testing of therapies, while organ chips replicate tumor microenvironments for personalized chemotherapy assessments. These tools have predicted patient responses with high accuracy in small cohorts, potentially reducing trial failures and accelerating drug development. However, scalability and integration into clinical workflows remain hurdles.

Clinical trials and regulatory approvals are expanding therapeutic options. The SANO-3 trial [53] investigates upfront immunotherapy in patients achieving clinical complete response (cCR) post-neoadjuvant therapy, aiming to defer surgery through active surveillance enhanced by nivolumab. Early results suggest non-inferior survival compared to standard resection. FDA approval of Tevimbra (tislelizumab-jsgr) in 2025 for first-line treatment of unresectable or metastatic esophageal squamous cell carcinoma (ESCC) marks a milestone, offering PD-1 inhibition with chemotherapy for PD-L1-positive tumors. Non-endoscopic screening via Cytosponge [54], a sponge-on-a-string device, detects Barrett’s esophagus and early EC with high acceptability, potentially shifting paradigms toward population-based screening and earlier preservation eligibility.

Looking to the future, AI-driven predictive models are enhancing cCR detection. Preliminary 2025 studies report algorithms achieving up to 98% sensitivity and 95% specificity in identifying high-risk ESCC, surpassing human performance. Integration could push accuracy beyond 90%, aiding de-escalation decisions. Proton therapy’s expansion is evidenced by the NRG-GI006 trial, a phase III comparison with intensity-modulated radiotherapy (IMRT), suggesting it as a cardiac-sparing standard in thoracic EC, with reduced mortality risks [55]. AI limitations include dataset biases; feasibility requires diverse training data, prioritizing over less mature CAR-T therapies.

Biomarker-driven de-escalation leverages ctDNA clearance as a surrogate endpoint. Extensions of the CheckMate 577 trial, which established adjuvant nivolumab’s benefit in resected EC, explore ctDNA for monitoring recurrence and therapy adjustment, potentially allowing reduced intensity in responders [56]. Bioengineered esophageal constructs, using scaffolds and regenerative techniques, address post-treatment strictures through customized grafts that promote tissue integration and function restoration. Advances in 3D bioprinting tailor these to patient anatomy, mitigating complications like anastomotic leaks [57]. Future research directions should first validate biomarkers, followed by regenerative medicine.

Global collaborations via organizations like the American Association for Cancer Research (AACR) and European Society for Medical Oncology (ESMO) are tackling immunotherapy resistance. Combination trials with chimeric antigen receptor T-cells (CAR-T) and oncolytic viruses aim to overcome solid tumor barriers, enhancing lytic and immunogenic effects [58]. Preclinical data suggest synergies that could boost organ preservation to 80% in responsive subtypes by 2030, though clinical translation requires addressing toxicity and resistance mechanisms. Controversies include equitable access and ethical considerations in AI and gene editing, with counterarguments emphasizing the need for diverse datasets to avoid biases.

To organize these advancements, the following table summarizes key areas, supporting evidence, and implications: Table 2.

## 6. Conclusions

Organ preservation in EC epitomizes patient-centered oncology, offering non-inferior survival and superior QoL compared to routine esophagectomy, especially in ESCC. Trials like SANO and emerging immunotherapy combinations validate its role, but challenges in assessment and toxicity demand refined protocols. Multidisciplinary approaches and maturing phase III data will likely establish organ-sparing as standard for suitable patients, potentially elevating global outcomes. With continued advancements in biomarkers and targeted modalities, organ preservation strategies are poised to become more inclusive, reducing the global burden of EC morbidity. This review’s novel framework underscores the need to address gaps in EAC and controversies in recurrence management for future progress.

## Figures and Tables

**Table 1:** Summary of Major Trials on Organ Preservation in Esophageal Cancer.

Trial Name	Design	Population	Intervention	Key Outcomes
CROSS (2012/2021) [6]	Phase III RCT	Resectable EC	nCRT + surgery vs. surgery	pCR 29%; 10-year OS 38% vs. 25%
preSANO (2018) [18]	Prospective cohort	Locally advanced EC	Multimodal response assessment post-nCRT	cCR accuracy 80%; guides surveillance
SANO (2025) [20]	Phase III RCT	Resectable EC	Active surveillance vs. immediate surgery post-nCRT	Non-inferior OS (2-year: 74% vs. 71%); organ preservation in 40–50%
CROC (2024) [19]	Phase II	Locally advanced EC	dCRT for responders	5-year OS 50%; viable preservation
ARMADILLO (JCOG1904, 2024) [32]	Phase III	ESCC post-ER	Adjuvant CRT	Locoregional control 93.4%; high preservation rates
ESOPEC (2016/2023) [33,34]	Phase III RCT	Resectable EAC	FLOT vs. CROSS nCRT	Improved OS with FLOT (66 vs. 57 months); better for EAC
JCOG1109 [35]	Phase III RCT	Locally advanced EC	Preoperative chemotherapy + surgery	3-year OS (NeoCF+D 72.1% vs. NeoCF 62.6% vs. NeoCF+RT 68.3%)
CheckMate 577 (2021/2025 follow-up) [36]	Phase III RCT	Resectable EC post-nCRT	Adjuvant nivolumab vs. placebo	DFS doubled (22.4 vs. 11 months); OS benefit in ESCC
NRG-GI006 (ongoing, 2025 interim) [15]	Phase II/III	Locally advanced EC	Proton vs. photon RT in nCRT	Reduced toxicities (20% lower); similar efficacy

Abbreviations: RCT = Randomized Controlled Trial; EC = Esophageal Cancer; ESCC = Esophageal Squamous Cell Carcinoma; EAC = Esophageal Adenocarcinoma; nCRT = Neoadjuvant Chemoradiotherapy; OS = Overall Survival; DFS = Disease-Free Survival; pCR = Pathologic Complete Response; cCR = Clinical Complete Response; NeoCF+D = Neoadjuvant Cisplatin/5-Fluorouracil/Docetaxel (also known as DCF regimen); NeoCF = Neoadjuvant Cisplatin/5-Fluorouracil (also known as CF regimen); NeoCF+RT = Neoadjuvant Cisplatin/5-Fluorouracil + Radiotherapy (also known as CF-RT regimen).

**Table 2:** Prioritized Future Directions.

Advancement Area	Key Supporting Evidence	Implications for Organ Preservation	Potential Challenges
Genomics (PD-L1, MSI)	Molecular profiling predicts response to ICIs; MSI-high subsets show improved outcomes.	Enables selection for non-surgical approaches in responsive patients.	Limited in low-PD-L1 tumors; requires broader genomic panels.
Biomarkers (ctDNA)	ctDNA dynamics correlate with OS/PFS; used for real-time monitoring.	Facilitates de-escalation and surveillance post-therapy.	Assay variability; not yet standard in all guidelines.
Proton Therapy	Reduces OAR doses and toxicities; NRG-GI006 supports cardiac-sparing.	Minimizes side effects, expanding eligibility for preservation.	High cost and limited centers; ongoing trials needed for OS data.
Hypofractionated Regimens	Safe in palliative care; comparable efficacy to CFRT.	Shorter courses improve patient compliance and quality of life.	Risk of late toxicities; integration with chemo requires caution.
Organoids/Organ Chips	Predict chemotherapy responses; mimic tumor microenvironment.	Personalized drug screening reduces ineffective treatments.	Scalability issues; ethical sourcing of patient tissues.
Clinical Trials (SANO-3, CheckMate 577)	Upfront immunotherapy in cCR; ctDNA as surrogate endpoint.	Defers surgery, improves DFS; allows therapy adjustment.	Patient selection critical; long-term OS data pending.
FDA Approvals (Tevimbra)	Approved for ESCC in 2025; combines with chemo.	Expands first-line options, enhancing preservation rates.	PD-L1 dependency; monitoring for immune-related AEs.
Screening Tools (Cytosponge)	Detects early lesions non-invasively.	Broadens early detection, increasing preservation candidates.	Biomarker specificity; integration into primary care.
AI Predictive Models	2025 studies show >90% accuracy in cCR/ESCC detection.	Improves diagnostic precision, aiding surveillance.	Data biases; need for explainable AI.
Bioengineered Constructs	Regenerative scaffolds prevent strictures.	Aids reconstruction post-treatment, reducing complications.	Biocompatibility; long-term integration studies needed.
Global Collaborations (AACR/ESMO)	Focus on resistance with CAR-T/oncolytic combos.	Potential 80% preservation by 2030 in subtypes.	Toxicity management; equitable global access.

Abbreviations: PD-L1 = Programmed Death-Ligand 1; MSI = Microsatellite Instability; ctDNA = Circulating Tumor DNA; OS = Overall Survival; PFS = Progression-Free Survival; cCR = Clinical Complete Response; ESCC = Esophageal Squamous Cell Carcinoma; CAR-T = Chimeric Antigen Receptor T-Cells; OAR = Organs at Risk; CFRT = Conventionally Fractionated Radiotherapy).

## Data Availability

No new data were created or analyzed in this study.
